# A Decade of Surgical Management of Renal Cell Carcinoma with IVC Thrombus and Bilateral Renal Tumors: Emphasis on Auto-transplantation

**DOI:** 10.15586/jkc.v12i2.367

**Published:** 2025-04-24

**Authors:** Abdul Rouf Khwaja, Aamir Mushtaq, Younis Mushtaq, Arif Hamid, Sajad Mali, Sajad Parra, Saqib Mehdi, Faheem ul Islam, Akil Lateif

**Affiliations:** Sher-i-Kashmir Institute of Medical Sciences (SKIMS), Main Road, Soura, Srinagar, Jammu and Kashmir, India

**Keywords:** Renal cell cancer, autotransplant, IVC thrombus

## Abstract

To assess the surgical outcomes and techniques in managing renal cell carcinoma (RCC) with inferior vena cava (IVC) thrombus and bilateral renal tumors with a focus on the role of autotransplantation in complex cases, this retrospective study analyzed 58 patients treated at our center between 2013 and 2023 for RCC with tumor thrombus extending into the IVC and, in some cases, the right atrium (RA). Surgical management included radical nephrectomy and thrombectomy with techniques adapted to thrombus level. For level I and II thrombi, innovative occluding maneuvers were used to control the contralateral renal vein. For level IV thrombi, a beating heart technique combined with cardiopulmonary bypass (CPB) was employed. Of the 10 patients with bilateral renal tumors, 2 underwent autotransplantation and 8 underwent bilateral partial nephrectomy. In this 10-year retrospective study of 58 patients with either RCC with venous tumor extension or bilateral RCC, 40 males and 18 females, with a mean age of 66 ± 8 years. Tumor involvement was predominantly right-sided (72.4%). Thrombus levels included 53.44% Level I, 25.9% Level II, and 3.4% Level IV. Intraoperative and postoperative complications were minimal, affecting 10 patients; patients with Level I thrombus had a better survival rate; and one patient with Level IV thrombus died postoperatively. The mean blood loss was 360 mL and the mean operative time was 195 minutes. Histopathology revealed clear cell carcinoma in 65.5% of cases. Among the 10 patients with bilateral renal tumors, autotransplantation and partial nephrectomies resulted in excellent renal preservation and favorable outcomes. This study demonstrates the effectiveness of radical nephrectomy and thrombectomy for RCC with venous tumor extension. Tailored surgical techniques, including autotransplantation for bilateral tumors, resulted in excellent outcomes with minimal complications. Personalized surgical strategies were key to preserving renal function and improving survival in complex RCC cases.

## Introduction

Considered one of the most lethal tumors affecting the renal system (1), renal cell carcinoma (RCC) accounts for 2–3% of all adult cancers. It encompasses various subtypes, each linked to distinct parts of the nephron and characterized by specific genetic and biological profiles (2, 3). A notable aspect of RCC is its propensity to extend intraluminally into the renal venous system, progressing cranially as venous tumor thrombosis, even reaching the right atrium (RA) or beyond (4). This venous invasion occurs in approximately 4–10% of RCC cases (4); fortunately, 45–70% of patients can be effectively treated with a combination of nephrectomy and thrombectomy (4).

In rare and complex cases, RCC can also manifest as bilateral renal tumors, often presenting additional surgical challenges, particularly when combined with venous tumor thrombus (5). These bilateral tumors necessitate careful surgical planning to preserve renal function while also achieving oncological control. In such cases, autotransplantation has emerged as a novel approach, allowing surgeons to perform ex vivo tumor resection with precise removal of the tumor and subsequent reimplantation of the kidney (6, 7). This strategy is particularly useful when dealing with bilateral tumors, where preserving kidney function is critical and where conventional in situ resection may be less feasible.

In patients initially diagnosed with a renal mass, symptoms such as lower extremity edema, dilated superficial abdominal veins, proteinuria, pulmonary embolism, isolated right-sided varicocele, and the presence of a right atrial mass may indicate the possibility of renocaval tumor extension (8). The complexity of surgery increases with the extent of thrombus extension, which is directly associated with higher morbidity and mortality rates (9). Managing these cases requires a multidisciplinary approach, presenting a significant challenge for urooncologists, especially when bilateral involvement or the need for advanced techniques such as autotransplantation is present.

## Materials and Methods

After obtaining approval from the institutional ethics committee, data of patients diagnosed with nonmetastatic renal tumors associated with tumor thrombus in the renal vein (RV) or inferior vena cava (IVC) and patients with bilateral renal tumors between 2013 and 2023 were collected. Given the retrospective nature of the study, informed consent was waived, after which the patients were analyzed with regards to their demographics (age/gender), tumor characteristics (laterality/level of thrombus), and intraoperative characteristics (blood loss/operative time). Outcomes (mortality/complications) were ascertained with a follow-up of up to 2 years in most patients.

## Results

A total of 58 patients diagnosed with RCC, accompanied by venous tumor extension or bilateral renal tumors, were included in this 10-year retrospective study. Of them, 48 patients who had RCC with tumor thrombus underwent radical nephrectomy and thrombectomy at our center, with surgical techniques tailored to the extent of tumor extension. Of the 10 patients who presented with bilateral renal tumors, 2 underwent radical nephrectomy on one side and ex vivo partial nephrectomy with bench dissection with autotransplantation on the other side and the remaining 8successfully underwent bilateral partial nephrectomy.

The patient cohort had a mean age of 66 ± 8 years, ranging from 17 to 71 years old. Of the 58 patients, 40 were males and 18 females, resulting in a male-to-female ratio of 2.2:1. Tumor involvement was more common on the right side, with 42 patients (72.4%) having right and 16 patients (27.6%) having left kidney tumors.

The distribution of thrombus levels showed that 31 patients (53.44%) had Level I thrombus, 15 (25.9%) had Level II thrombus, and 2 (3.4%) had Level IV thrombus extension; no patients had Level III thrombus extension.

Intraoperative and major postoperative complications were minimal, with 48 of the 58 patients experiencing no significant complications; however, 10 patients encountered complications where one patient (1.72%) with Level IV thrombus succumbed postsurgery. The average blood loss during surgery was 360 mL, ranging from 200 to 1000 mL, and the mean operative time was 195 minutes, ranging from 180 to 345 minutes.

Histopathological analysis revealed that clear cell carcinoma was the most common subtype, found in 38 cases (65.5%), followed by papillary carcinoma in 10 cases (17.2%), chromophobe carcinoma in 6 cases (10.3%), and squamous cell carcinoma in 4 cases (6.9%). Over a follow-up period of 7 years ± 3 months, patients with Level I thrombus showed better survival rates compared to those with Level II thrombus.

Among the 10 patients with bilateral renal tumors, 2 patients who underwent partial nephrectomy with autotransplantation demonstrated excellent postsurgery renal function preservation and the 8 who underwent bilateral partial nephrectomy also had favorable outcomes. Overall, complications in the cohort included one perioperative death, three cases of deep vein thrombosis, and seven cases of wound infection. The results are presented in Table 1.

**Table 1:** Summary of results.

Total Patients	58
Mean Age	66 ± 8 years (range: 17–71)
Gender Distribution	Male: 40 (69%) Female: 18 (31%)
Male-to-Female Ratio	2.2:1
Tumor Laterality	Right kidney: 42 (72.4%) Left kidney: 16 (27.6%) Bilateral renal tumors: 10 patients (17.2%)
Mean Blood Loss	360 mL (range: 200–1000 mL)
Mean Operative Time	195 minutes (range: 180–345 minutes)
Perioperative Death	1 patient (1.72%) level IV thrombus
Complications	10 patients with complications (17.2%)
Specific Complications	Per operative death: 1 Deep vein thrombosis: 3 Wound infection: 7
Histopathological Findings	Clear cell carcinoma: 38 (65.5%) Papillary carcinoma: 10 (17.2%) Chromophobe carcinoma: 6 (10.3%) Squamous cell carcinoma: 4 (6.9%)

For most patients, follow-up data of up to 2 years was available, and all patients had stable renal function on follow-up. None had developed any recurrence or metastasis during the same period.

### Surgical techniques for RCC thrombus

#### Focus on autotransplantation

**Figure 1:**
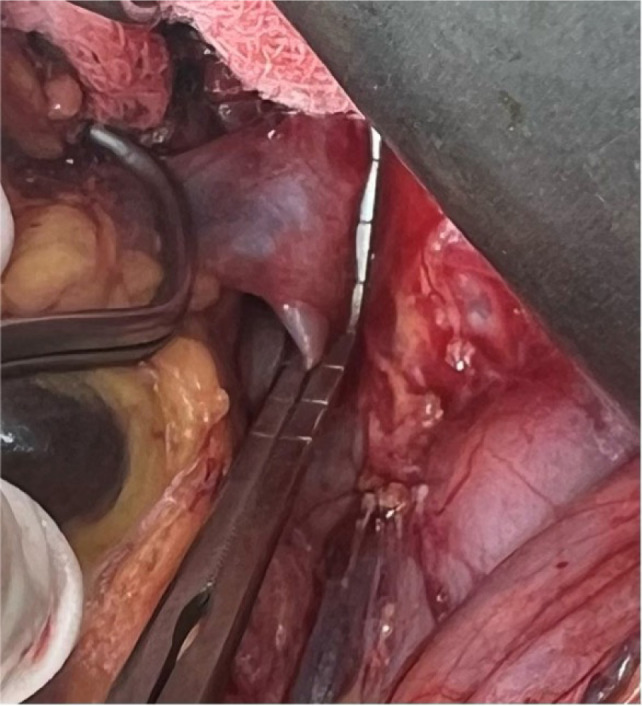
Intraoperative view of Level I thrombus in a patient with RCC showing side clamp in place.

**Figure 2:**
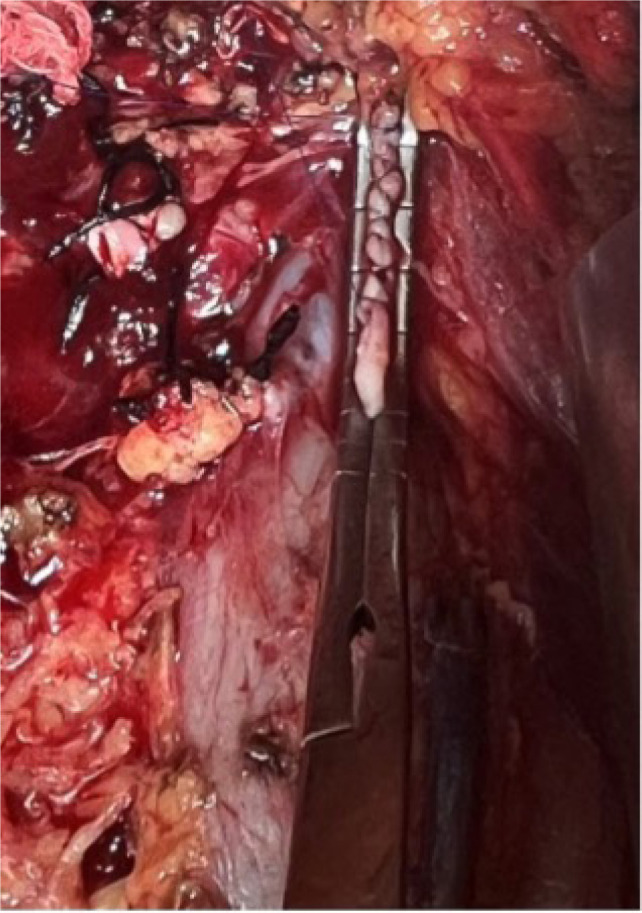
Intraoperative view showing closure of inferior vena cava following removal of a Level I thrombus.

**Figure 3:**
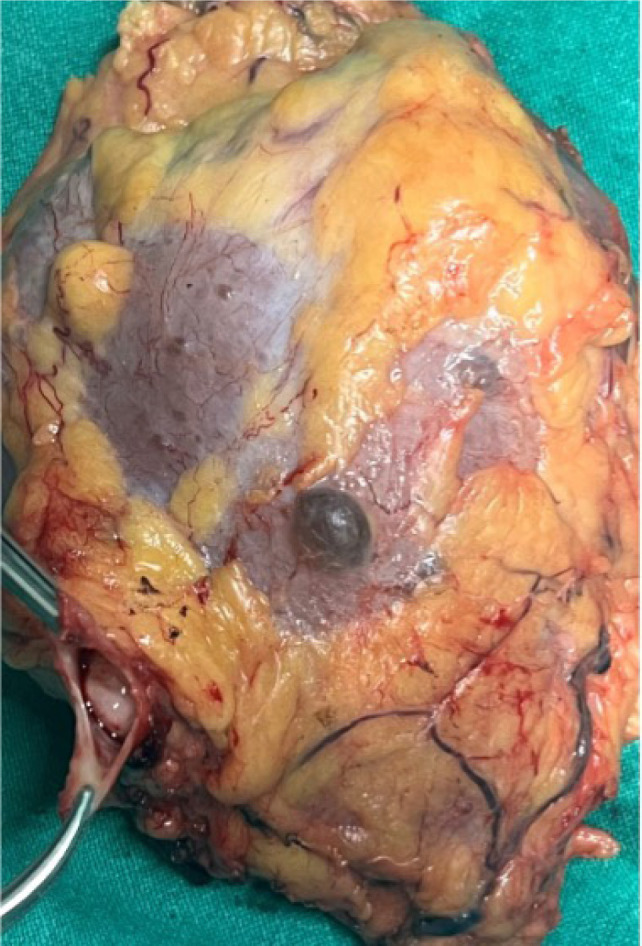
Radical nephrectomy specimen showing a tumor thrombus in the renal vein.

The surgical approach for thrombus extension in our study was adapted based on the level of involvement. For patients with a Level I thrombus, vascular side clamps were applied without obstructing the IVC inflow (Figure 1). Following removal of the thrombus, the IVC defect was closed using a continuous 4-0 Prolene suture (Figure 2). Figure 3 shows the radical nephrectomy specimen with tumor thrombus in the RV.

In cases involving Level II thrombus, extensive mobilization of the IVC was required, which included gaining both proximal and distal control of the IVC as well as ligating and dividing the lumbar veins to minimize blood loss. To extract the thrombus, vascular clamps were applied. For right-sided renal tumors, the left RV was clamped, and for left-sided renal tumors, the right renal artery was clamped to ensure control. The cavotomy was then repaired using a continuous 4-0 Prolene suture.

**Figure 4:**
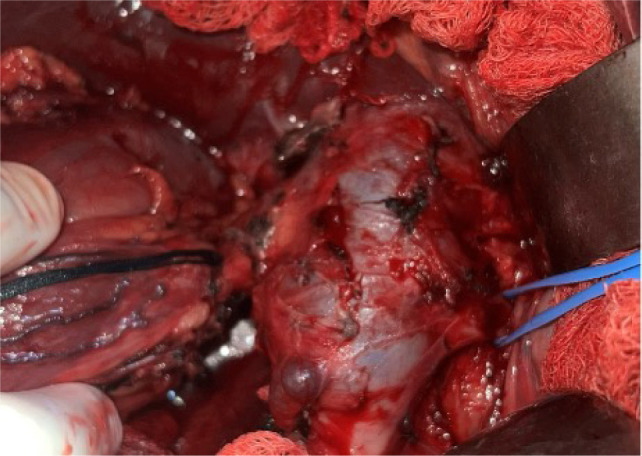
Notable bulging of the inferior vena cava due to the presence of a large thrombus with the contralateral vein appropriately controlled during the procedure.

**Figure 5:**
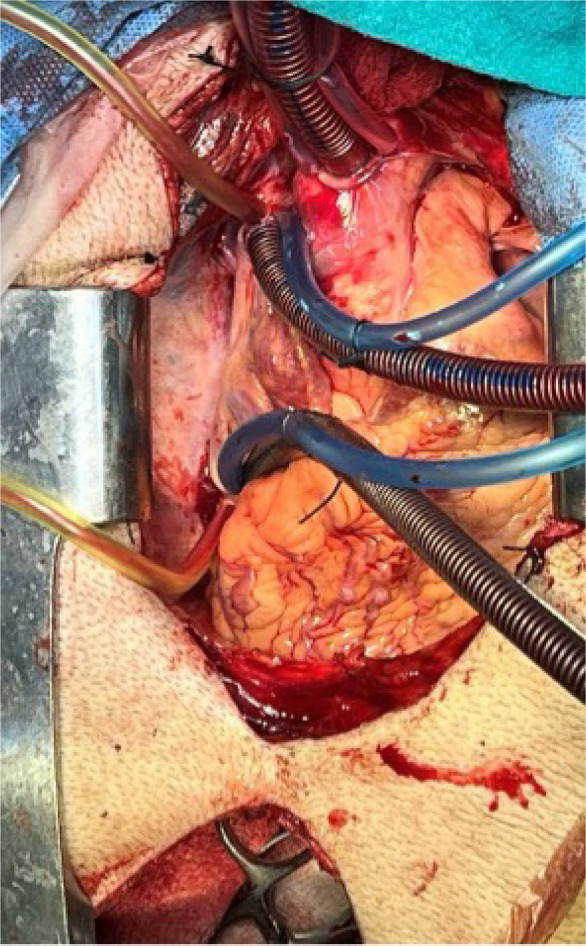
Sternotomy performed with vascular control of the inferior vena cava, superior vena cava, and right atrium in a patient with Level IV thrombus. Lower portion of the image shows the nephrectomy incision.

**Figure 6:**
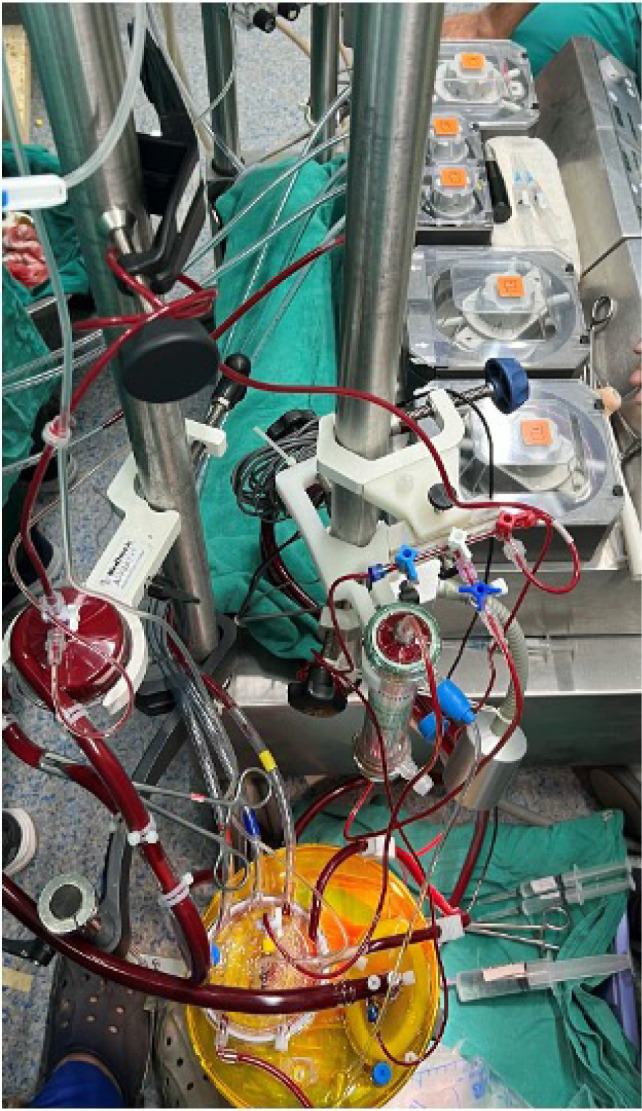
Cardiopulmonary bypass setup used for the resection of Level IV thrombus.

Two patients presented with RCC and tumor thrombus extending into the IVC (supradiaphragmatic IVC). Both cases were managed surgically in the Cardiovascular and Thoracic Surgery (CVTS) operating theater. The procedures involved a median sternotomy. The thrombus was carefully controlled and extracted from the suprahepatic IVC under direct visualization, with meticulous attention to detail to prevent embolization (Figures 4–6).

#### Bench dissection and autotransplantation

**Figure 7:**
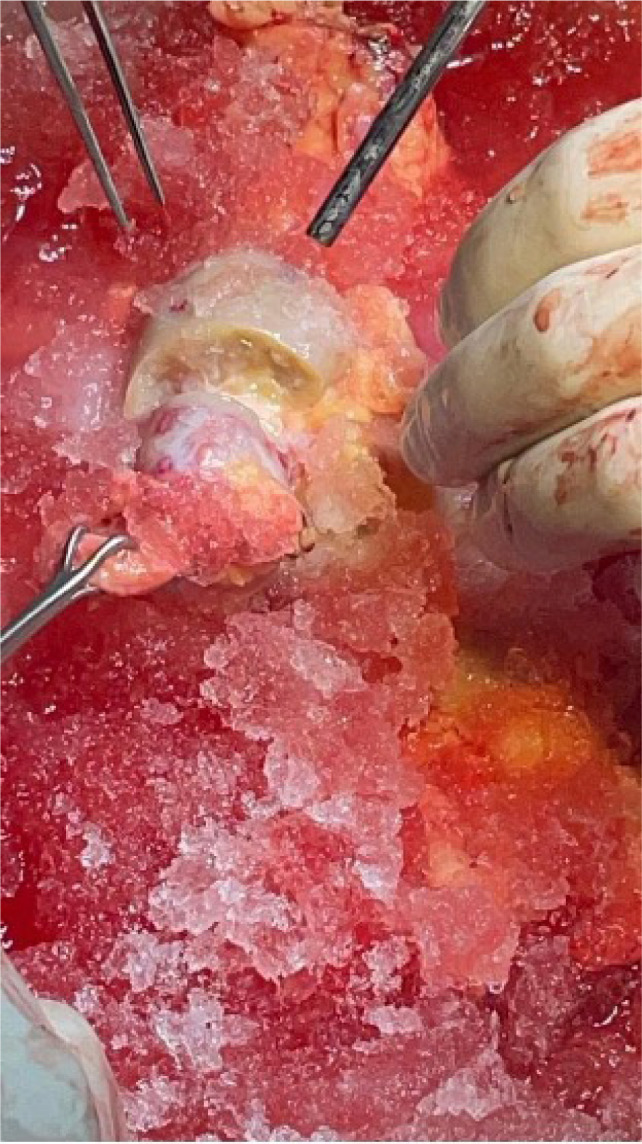
Intraoperative image depicting bench dissection where the kidney is carefully prepared ex vivo prior to autotransplantation. The dissection allows for precise removal of the tumor while preserving maximum healthy renal tissue.

**Figure 8:**
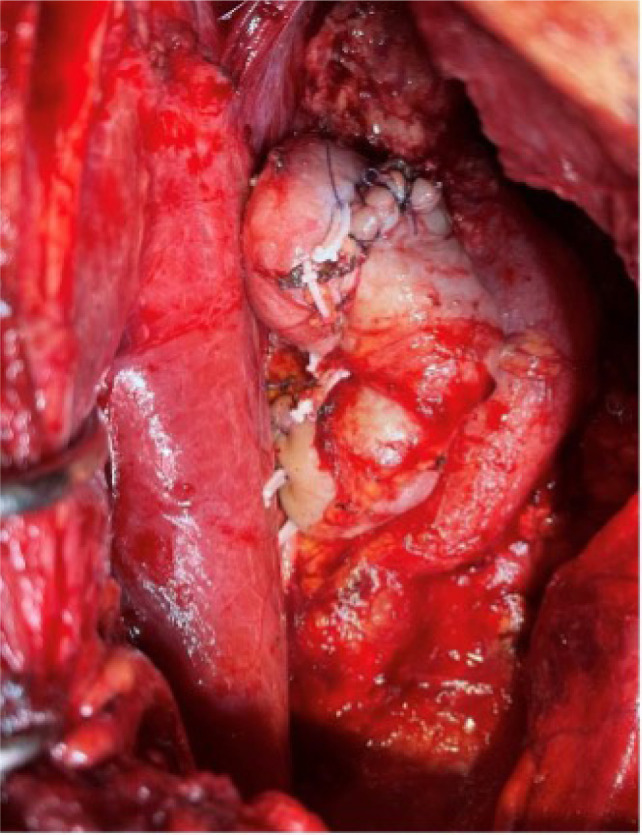
Intraoperative kidney depicting the autotransplanted kidney.

**Figure 9:**
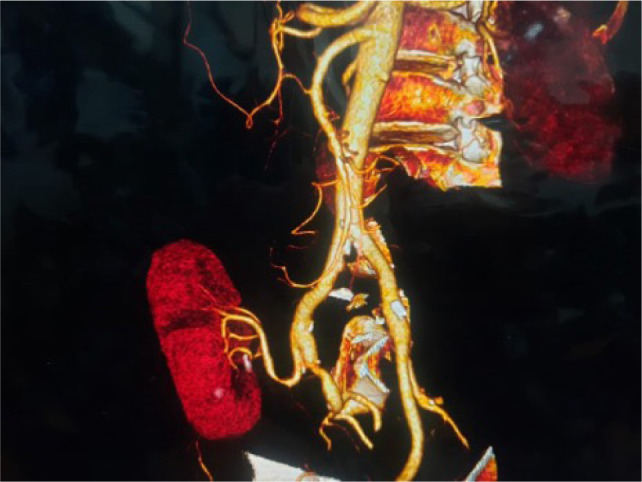
Three-dimensional reconstruction of computed tomography angiogram post autotransplantation displaying successfully transplanted kidney with clear visualization of the vascular anastomosis.

The endophytic tumor was identified on imaging and verified through intraoperative ultrasound, where it was carefully excised with a margin of normal tissue and hemostasis ensured with bipolar cautery. The renal parenchyma was closed using the interrupted 4-0 Vicryl (Figures 7 and 8). The renal artery and vein were cleaned, trimmed, and prepared for reanastomosis. A retroperitoneal approach was chosen for autotransplantation, with the patient now placed supine. The external iliac artery and vein were identified and dissected free for vascular anastomosis. The RV was anastomosed to the external iliac vein using running 5-0 Prolene sutures. Next, the renal artery was anastomosed to the external iliac artery using 6-0 Prolene in a continuous fashion, ensuring no tension or kinks in the vessels (Figure 9). After completing the anastomoses, clamps were removed and the kidney was reperfused. Immediate capillary refill and healthy color change confirmed adequate perfusion. Doppler ultrasound was used intraoperatively to confirm the presence of good arterial flow and venous drainage. The ureter was reimplanted into the bladder using a modified Lich-Gregoir technique with 4-0 Polydioxanone Sutures (PDS’) to create a tension-free, watertight anastomosis. Meticulous hemostasis was achieved throughout the procedure using absorbable hemostatic agents (e.g., Surgicel). The retroperitoneum was closed using 2-0 Vicryl sutures and the skin was closed with subcuticular 4-0 Monocryl sutures.

The patient was closely monitored in the intensive care unit (ICU) for 48 hours postoperatively, with regular Doppler ultrasound assessments to confirm ongoing graft function. Immunosuppressive therapy was not initiated as the kidney was autotransplanted. Postoperative creatinine and urine output remained stable, confirming successful renal function preservation. This operative approach allowed for oncological control on one side while simultaneously preserving renal function on the other through ex vivo tumor resection and autotransplantation.

## Discussion

This study provides valuable insights into the surgical management of RCC and venous tumor extension, which offer a significant contribution to the ongoing discourse on the optimal approaches to nephrectomy and thrombectomy in the presence of venous tumor thrombi, particularly in patients with bilateral renal tumors.

The patient cohort had a mean age of 66 ± 8 years, which aligns with the typical age distribution for RCC, a malignancy primarily affecting older adults (2). The male-to-female ratio of 2.2:1 is consistent with previous literature, which reports a higher incidence of RCC in men than in women (2). Tumor involvement was predominantly on the right side (72.4%), reflecting the known tendency of RCC to more frequently affect the right kidney, possibly due to anatomical or embryological factors.

The distribution of thrombus levels in this study provides crucial insights into the progression and surgical complexity of RCC with venous tumor extension. The majority of patients (53.44%) had Level I thrombus, followed by 25.9% with Level II thrombus, and a small percentage (3.4%) with Level IV thrombus. Previous studies, such as those by Blute et al. and Klatte et al., have demonstrated that the prognosis worsens with increasing thrombus level, largely due to the increased surgical complexity and the risk of complications (4, 9). In our cohort, the higher rates of survival among patients with Level I thrombus compared to those with Level II thrombus are consistent with these findings.

The surgical outcomes in our study are encouraging. Intraoperative and major postoperative complications were minimal, with 48 of the 58 patients experiencing no significant complications. This complication rate compares favorably with prior studies, where rates of major complications ranged from 20% to 40% (10). The average blood loss of 360 mL and mean operative time of 195 minutes are within the range reported in the literature, indicating that the surgical techniques employed were efficient and well executed. However, the death of a patient with Level IV thrombus postsurgery underscores the high risk associated with advanced thrombus levels. Previous studies have similarly shown that patients with extensive thrombus, particularly Level IV, face significant perioperative mortality due to the complexity of the procedure and the hemodynamic challenges associated with thrombus extension into the IVC or RA.

Histopathological analysis revealed clear cell carcinoma as the predominant subtype (65.5%), followed by papillary, chromophobe, and squamous cell carcinoma. These findings align with global data on RCC, where clear cell carcinoma constitutes approximately 70–80% of cases (2). The survival advantage observed in patients with clear cell carcinoma and Level I thrombus further supports the notion that early detection and intervention can significantly improve outcomes in this patient population (9).

Neoadjuvant therapy using a combination of targeted therapy and immunotherapy has also been described for the treatment of patients with RCC with tumor thrombus. While Gu et al. reported that neoadjuvant therapy is safe and feasible with acceptable perioperative outcomes in RCC with tumor thrombus, Cai et al. reported that neoadjuvant therapy of sorafenib or sunitinib might not improve survival outcomes for high-risk RCC patients with tumor thrombus (11, 12). Stereotactic body radiotherapy (SBRT) has also been described as a potentially safe treatment option in the unresectable setting for RCC patients with IVC tumor thrombus (13). No patients in our study received neoadjuvant therapy or SBRT.

One of the most noteworthy aspects of our study is the successful management of bilateral renal tumors in 10 patients. The two patients who underwent radical nephrectomy on one side and bench dissection with autotransplantation on the other side demonstrated excellent renal function preservation postsurgery. This novel approach allowed for precise tumor resection and subsequent renal reimplantation, thereby optimizing oncological outcomes while maintaining renal function. These findings are consistent with other series, such as those by Metwalli et al., where autotransplantation has been proven to be a viable option in complex cases involving bilateral tumors (5). Novick et al. pioneered the application of autotransplantation for RCC with complex or bilateral tumours, highlighting its role in preserving renal function (14). Medina et al. described kidney autotransplantation as a potential treatment for complex renovascular, ureteral, or neoplastic conditions (15). They highlighted that autotransplantation can be a valuable approach for complex cases in the absence of other therapeutic options. The eight patients in our series who underwent bilateral partial nephrectomies also had favorable outcomes, with no significant decline in renal function, further supporting the role of nephron-sparing surgery in selected cases of bilateral RCC.

The overall complication rate in our cohort was low, with one perioperative death, three cases of deep vein thrombosis, and seven cases of wound infection. These complication rates are similar to those reported in the literature where perioperative mortality for RCC with venous thrombus ranges from 1% to 5% and nonlethal complications occur from 10% to 30% of cases (4). Our findings suggest that with careful preoperative planning, skilled surgical techniques, and a multidisciplinary approach, the risks associated with these complex surgeries can be minimized.

In conclusion, our study adds to the growing body of evidence that supports radical nephrectomy and thrombectomy as the standard of care for RCC with venous tumor thrombus. The use of advanced techniques, such as bench dissection with autotransplantation in cases of bilateral renal tumors, offers a promising option for preserving renal function while simultaneously achieving oncological control. The outcomes from our cohort reinforce the importance of individualized surgical planning and the need for further research to refine these techniques and improve patient outcomes.

## Conclusion

This study underscores the effectiveness of radical nephrectomy and thrombectomy in managing RCC with venous tumor extension. Tailored surgical techniques, including the innovative use of autotransplantation for bilateral tumors, led to excellent outcomes with minimal complications. Clear cell carcinoma predominated, and patients with lower level thrombus demonstrated superior survival rates. These findings highlight the power of personalized surgical strategies in enhancing survival and preserving renal function, offering a promising outlook for complex RCC cases.
